# Development and Validation of a Scoring System for Early Diagnosis of Malignant Pleural Effusion Based on a Nomogram

**DOI:** 10.3389/fonc.2021.775079

**Published:** 2021-12-07

**Authors:** Aihua Wu, Zhigang Liang, Songbo Yuan, Shanshan Wang, Weidong Peng, Yijun Mo, Jing Yang, Yanqing Liu

**Affiliations:** ^1^ Department of Laboratory Medicine, Ningbo First Hospital, Ningbo, China; ^2^ Department of Thoracic Surgery, Ningbo First Hospital, Ningbo, China; ^3^ Department of Clinical Laboratory, The Affiliated People Hospital of Ningbo University, Ningbo, China; ^4^ Department of Respiratory and Critical Care Medicine, The Affiliated People Hospital of Ningbo University, Ningbo, China; ^5^ Department of Respiratory and Critical Care Medicine, Ningbo First Hospital, Ningbo, China

**Keywords:** malignant pleural effusion, scoring system, prediction model, nomogram, tuberculous pleurisy effusion

## Abstract

**Background:**

The diagnostic value of clinical and laboratory features to differentiate between malignant pleural effusion (MPE) and benign pleural effusion (BPE) has not yet been established.

**Objectives:**

The present study aimed to develop and validate the diagnostic accuracy of a scoring system based on a nomogram to distinguish MPE from BPE.

**Methods:**

A total of 1,239 eligible patients with PE were recruited in this study and randomly divided into a training set and an internal validation set at a ratio of 7:3. Logistic regression analysis was performed in the training set, and a nomogram was developed using selected predictors. The diagnostic accuracy of an innovative scoring system based on the nomogram was established and validated in the training, internal validation, and external validation sets (*n* = 217). The discriminatory power and the calibration and clinical values of the prediction model were evaluated.

**Results:**

Seven variables [effusion carcinoembryonic antigen (CEA), effusion adenosine deaminase (ADA), erythrocyte sedimentation rate (ESR), PE/serum CEA ratio (CEA ratio), effusion carbohydrate antigen 19-9 (CA19-9), effusion cytokeratin 19 fragment (CYFRA 21-1), and serum lactate dehydrogenase (LDH)/effusion ADA ratio (cancer ratio, CR)] were validated and used to develop a nomogram. The prediction model showed both good discrimination and calibration capabilities for all sets. A scoring system was established based on the nomogram scores to distinguish MPE from BPE. The scoring system showed favorable diagnostic performance in the training set [area under the curve (AUC) = 0.955, 95% confidence interval (CI) = 0.942–0.968], the internal validation set (AUC = 0.952, 95% CI = 0.932–0.973), and the external validation set (AUC = 0.973, 95% CI = 0.956–0.990). In addition, the scoring system achieved satisfactory discriminative abilities at separating lung cancer-associated MPE from tuberculous pleurisy effusion (TPE) in the combined training and validation sets.

**Conclusions:**

The present study developed and validated a scoring system based on seven parameters. The scoring system exhibited a reliable diagnostic performance in distinguishing MPE from BPE and might guide clinical decision-making.

## Introduction

Pleural effusion (PE) is a common clinical problem resulting from increased fluid in the pleural cavity (1, 2). The various etiologies of PE can be divided into benign pleural effusion (BPE) and malignant pleural effusion (MPE) (3). In China, the majority of BPE arises from tuberculous pleural effusion (TPE), parapneumonic effusion (PPE), and heart failure (HF) (4). With regard to MPE, lung cancer, breast cancer, and lymphomas account for over 75% of MPE cases (1, 2, 5). The presence of MPE indicates systemic cancer dissemination and a reduction of life expectancy and quality in patients (6). The median survival in patients with MPE is 3–12 months (7, 8). Hence, an accurate and noninvasive method to diagnose patients with MPE is crucial for therapeutic decisions.

The initial diagnostic approaches to differentiate between MPE and BPE include PE cytology, closed pleural biopsy, and thoracoscopy (9–11). PE cytology is a standard method of MPE diagnosis with high specificity, while the sensitivity is only about 60%, depending on factors such as tumor type, stage of primary malignancy, and expertise of the pathologist (12). Similarly, closed pleural biopsy is an invasive method with some complications, and its diagnostic yield is only 50%–70% (13). Thoracoscopy is an efficient tool with a highly sensitive diagnostic accuracy for patients with MPE (14). However, due to its high cost, it may not be available at all facilities. Additionally, some patients with poor performance status (>2) and inexperience of the operator might limit the application of this intervention (15). Therefore, developing a cost-effective and convenient diagnostic tool is crucial for clinical practice.

Several biomarkers have been investigated for the identification of MPE and BPE in previous studies, such as adenosine deaminase (ADA), carcinoembryonic antigen (CEA), and lactate dehydrogenase (LDH) (16–18). However, no single marker from blood or PE could obtain both high sensitivity and specificity. Many studies have indicated that a combination of two or more biomarkers could improve the diagnostic performance in patients with MPE (19, 20). Reportedly, several studies have revealed that the PE/serum ratio of several tumor markers has higher diagnostic accuracy than a single index for MPE and BPE (17, 21). However, most of the published studies are single-center, with limited sample sizes, and lack validation.

Therefore, the present study aimed to construct and validate the efficiency of a nomogram with multiple parameters to distinguish between MPE and BPE. Next, we aimed to create a novel predictive scoring system based on the nomogram for clinical application. The scoring system was applied to differentiate between lung cancer-associated MPE and TPE and assess the diagnostic performance of the two most common exudative effusions diseases.

## Materials and Methods

### Study Population

**Figure 1:**
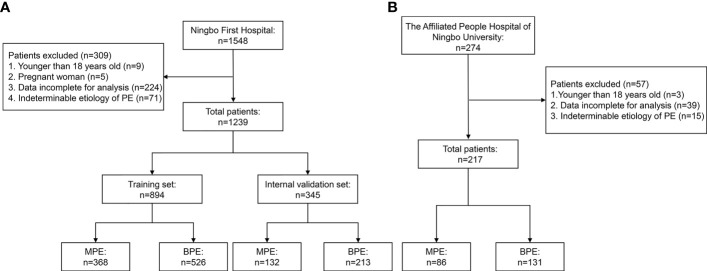
Flowchart of the study participants. **(A)** Ningbo First Hospital set. **(B)** The Affiliated People Hospital of Ningbo University set.

The clinical data of patients with PE diagnosed and who underwent medical treatment at the Ningbo First Hospital between January 2014 and December 2020 were collected retrospectively. The study flowchart is shown in **Figure 1A**. A total of 1,239 consecutive patients fulfilled the inclusion criteria. Patients were randomly divided into a training set and an internal validation set at a ratio of 7:3. From June 2020 to June 2021, 217 patients with PE from the Affiliated People Hospital of Ningbo University comprised the external validation set. The inclusion criteria were as follows: a) PE was diagnosed after ultrasonography, chest CT, or X-ray and b) patients underwent diagnosis for MPE or BPE by cytology and/or thoracentesis and/or pleural biopsy and follow-up (at least 6 months). The exclusion criteria were as follows: a) patients <18 years old; b) pregnant women; c) data incomplete for analysis; d) and indeterminable etiology of PE. This study was approved by the Institutional Ethics Committee and was conducted in accordance with the Helsinki Declaration. The requirement for written informed consent was waived based on the retrospective nature.

### Diagnostic Criteria

MPE was defined by the presence of malignant cells in PE cytology and cell block together with immunohistochemistry or pleural biopsy. The criteria for BPE were as follows: a) no tumor cells found in PE; b) PE of a known etiology, such as TPE or parapneumonic PE, that vanished after optimal treatment; and c) no signs of malignant disease were established during the follow-up. TPE was defined by acid-fast stains or Lowenstein–Jensen cultures of PE, sputum, and bronchoalveolar lavage fluid or the presence of pleural caseating granulomas. Parapneumonic PE was defined as PE associated with bacterial pneumonia, which disappeared after antibiotic therapy. In addition, other types of MPE were diagnosed based on well-established diagnostic criteria.

### Data Collection

Demographic, clinical, and laboratory variables, including age, sex, smoking history, levels of tumor biomarkers [CEA, cytokeratin 19 fragment (CYFRA 21-1), carbohydrate antigen 125 (CA125), carbohydrate antigen 19-9 (CA19-9), and neuron-specific enolase (NSE)] in PE and serum, blood routine [white blood cells (WBC), lymphocytes (LC), and neutrophil cells (NC)], effusion routine [effusion WBC, percentage of lymphocytes (L%), and percentage of neutrophil (N%)], effusion biochemical parameters [(ADA, LDH, total protein (TP), and glucose (Glu)], and serum biochemical parameters [erythrocyte sedimentation rate (ESR), C-reactive protein (CRP), LDH, and ADA] were collected. Optimal cutoff values were obtained using the Youden index. All laboratory variables and ratios were transformed into categorical variables based on the optimal cutoff values.

### Statistical Analysis

Continuous variables are expressed as the mean ± SD and compared using either a *t*-test or the Mann–Whitney *U* test, as appropriate. Categorical variables were compared using the chi-square test or Fisher’s exact test. Univariate logistic regression analysis was applied to investigate the independent risk factors in the training set, and all variables at a significant level [area under the curve (AUC) >0.65] were candidates for multivariate analysis. Then, stepwise selection using the Akaike information criterion (AIC) in the multivariable regression models identified the statistically significant variables. Odds ratios (ORs) were estimated and presented with their 95% confidence intervals (CIs). Selected variables were incorporated into the nomograms to construct the scoring system using the rms package of R (version 4.0.5). Calibration curves were measured with 1,000 bootstrap resamples by plotting the observed probability *vs.* the nomogram-predicted probability. Decision curve analysis (DCA) was performed to determine the clinical value of the prediction model by calculating the net benefits at various threshold probabilities in the validation set. Receiver operating characteristic (ROC) curves and the corresponding AUCs were calculated to determine the discrimination capacity of the models while predicting MPE. Moreover, the sensitivity, specificity, predictive values, and likelihood ratios were determined to assess the diagnostic accuracy of the nomogram. All statistical analyses were performed using R (packages rms, MASS, OptimalCutpoints, pROC, and rmda) and SPSS (version 22.0). All statistical tests were two-sided, and a *p* < 0.05 was deemed significant.

## Results

### Clinical Characteristics

The flowchart of the patient inclusion and exclusion process in this study is presented in **Figure 1**. A total of 1,239 eligible patients from Ningbo First Hospital were reviewed and randomly divided into the training set (*n* = 894) and the internal validation set (*n* = 345). Furthermore, 217 eligible patients from The Affiliated People Hospital of Ningbo University comprised the external validation set. The demographic and clinical characteristics in the training and validation sets are shown in **Table 1**. No significant differences were observed in age, gender, and smoking history between the training and validation sets. In the present study, the most common etiology of MPE was lung cancer, while TPE was the leading cause of BPE.

**Table 1:** Baseline characteristics of the study set.

Parameters	Training set (*n* = 894)	Internal validation set (*n* = 345)	External validation set (*n* = 217)
Age (years)	60.7 ± 18.4	61.1 ± 18.3	62.2 ± 19.5
Gender			
Male	573 (64.1%)	215 (62.3%)	144 (66.4%)
Female	321 (35.9%)	130 (37.9%)	73 (33.6%)
Smoking history			
Yes	336 (37.6%)	132 (38.3%)	81 (37.3%)
No	558 (62.4%)	213 (61.7%)	136 (62.7%)
MPE			
Lung cancer	281 (31.4%)	100 (29.0%)	67 (30.9%)
Breast cancer	9 (1%)	8 (2.3%)	4 (1.8%)
Ovarian cancer	9 (1%)	3 (0.9%)	2 (0.9%)
Lymphoma	12 (1.3%)	2 (0.6%)	2 (0.9%)
Mesothelioma	7 (0.8%)	4 (1.2%)	2 (0.9%)
Other cancers	50 (5.6%)	15 (4.3%)	9 (4.1%)
BPE			
Tuberculous pleurisy	265 (29.6%)	96 (27.8%)	66 (30.4%)
Parapneumonic effusion	85 (9.5%)	35 (10.1%)	22 (10.1%)
Parasitic infection	20 (2.2%)	8 (2.3%)	7 (3.2%)
Empyema	44 (4.9%)	21 (6.1%)	10 (4.6%)
Heart failure	71 (7.9%)	34 (9.9%)	17 (7.8%)
Other benign diseases	41 (4.6%)	19 (5.5%)	9 (4.1%)

Other cancers: colorectal cancer, gastric cancer, esophageal cancer, prostate cancer, renal cell carcinoma, endometrial cancer, liver cancer, cervical cancer, nasopharyngeal cancer, pancreatic cancer, thyroid cancer. Other benign diseases: nephrotic syndrome, systemic lupus erythematosus, hydropneumothorax, cirrhosis, interstitial lung disease, pericardial disease, diabetic nephropathy, sicca syndrome, chylothorax, hyperthyroidism.

MPE, malignant pleural effusion; BPE, benign pleural effusion.

### Development and Validation of the Prediction Model

**Figure 2:**
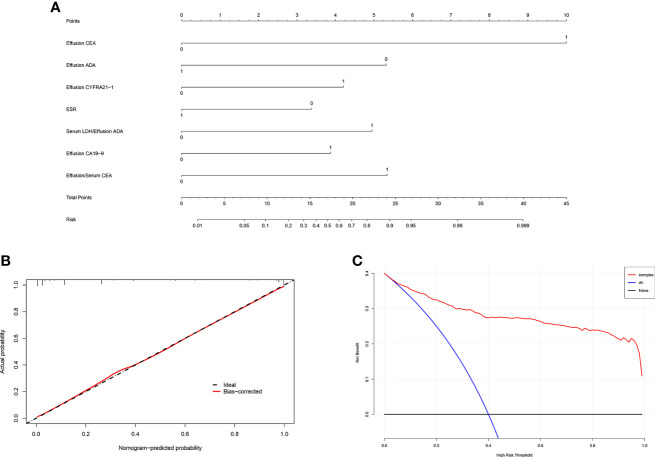
Development and validation of the diagnostic nomogram. **(A)** Nomogram was developed to differentiate between malignant pleural effusion (MPE) and benign pleural effusion (BPE) in the training set. **(B)** Calibration curve of the nomogram. **(C)** Clinical usefulness of the diagnostic nomogram.

All variables used in the analysis were based on the data obtained before treatment. In the training set, statistically significant differences were observed in age, gender, and most laboratory variables between the MPE and BPE groups (**Supplementary Table S1**). The results of the univariate logistic analysis are presented in **Supplementary Table S2**. A total of 29 variables showed statistical significance, of which 19 had an AUC > 0.6. To establish an accurate prediction model, 12 variables with an AUC > 0.65 were subjected to multivariate regression analysis. Then, stepwise selection using AIC in the regression modeling identified seven variables discriminating MPE from BPE at the highest order. They were as follows: ESR (OR = 0.36, 95% CI = 0.221–0.579), effusion CEA (OR = 20.51, 95% CI = 10.24–44.44), effusion CA19-9 (OR = 3.218, 95% CI = 1.737–6.042), effusion CYFRA21-1 (OR = 3.56, 95% CI = 2.074–6.177), PF/serum CEA ratio (CEA ratio; OR = 5.019, 95% CI = 2.97–8.577), effusion ADA (OR = 0.2, 95% CI = 0.071–0.535), and serum LDH/effusion ADA ratio (cancer ratio, CR; OR = 4.458, 95% CI = 1.738–11.86) (**Table 2**). The prediction model that incorporated the above independent variables was developed and presented as a nomogram (**Figure 2A**). The calibration curve of the nomogram showed that there was good agreement between the prediction and observation results in the training set (**Figure 2B**). In the DCA, this diagnostic nomogram offered a net benefit over the “treat-all” or “treat-none” strategy at a threshold probability >3%, which indicated that the nomogram had favorable clinical utility (**Figure 2C**). Moreover, the nomogram yielded an AUC of 0.955 (95% CI = 0.942–0.968) in the training set with satisfactory discrimination and calibration in the internal validation set (AUC = 0.953, 95% CI = 0.933–0.974) and external validation set (AUC = 0.968, 95% CI = 0.944–0.991) (**Supplementary Figures S1A–D**).

**Table 2:** Multivariate logistic regression analysis of the clinical parameters in the training set.

Variable	OR (95% CI)	*p*-value
ESR, >43 *vs*. ≤43 mm/h	0.36 (0.221–0.579)	<0.0001*
Effusion CEA, >5 *vs*. ≤5 ng/ml	20.51 (10.24–44.44)	<0.0001*
Effusion CA19-9, >9.2 *vs*. ≤9.2 ng/ml	3.218 (1.737–6.042)	0.0002*
Effusion CYFRA21-1, >59.6 *vs*. ≤59.6 ng/ml	3.56 (2.074–6.177)	<0.0001*
CEA ratio, >1.14 *vs*. ≤1.14	5.019 (2.97–8.577)	<0.0001*
Effusion ADA, >25 *vs*. ≤25 U/L	0.2 (0.071–0.535)	0.0018*
CR, >9.4 *vs*. ≤9.4	4.458 (1.738–11.86)	0.0023*

CRP, C-reactive protein; ADA, adenosine deaminase; ESR, erythrocyte sedimentation rate; CEA, serum carcinoembryonic antigen; CA19-9, carbohydrate antigen 19-9; CYFRA21-1, cytokeratin 19 fragment; CR, serum LDH/effusion ADA; CEA ratio, effusion/serum CEA; OR, odds ratio; AUC, area under curve; CI, confidence interval.

*p < 0.05.

### Diagnostic Performance of the Scoring System Based on the Nomogram Scores

**Figure 3:**
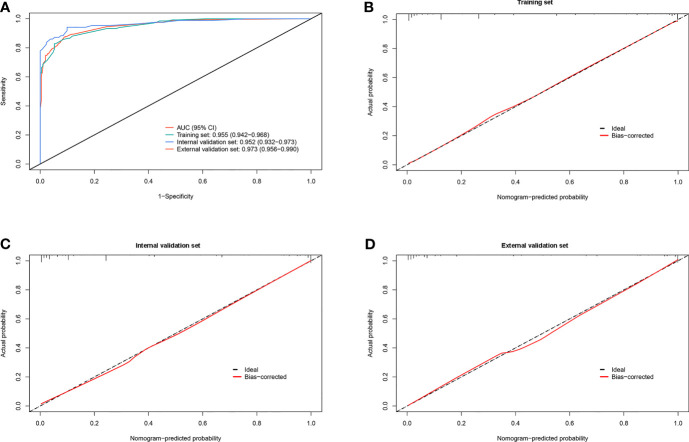
Diagnostic ability and calibration of the scoring system for identifying malignant pleural effusion (MPE) from benign pleural effusion (BPE). **(A)** Plot showing the receiver operating characteristic (ROC) curves of the scoring system in the training, internal validation, and external validation sets. **(B–D)** Plots presenting the calibration curves of the scoring system in the training, internal validation, and external validation sets.

In the training set, effusion CEA exhibited the largest impact on the separation of MPE from BPE in the model and was determined as 10 points. Sequentially, the remaining six variables were assigned scores as follows: ESR (3 points), effusion CA19-9 (4 points), effusion CYFRA21-1 (4 points), CEA ratio (5 points), effusion ADA (5 points), and CR (5 points). The total high points based on the sum of the defined points for each variable in the nomogram could be diagnosed as MPE (**Table 3**). The optimal cutoff values for the total scores (range = 0–36) were classified based on the ROC analysis. When the cutoff value was 15, the corresponding sensitivity, specificity, positive likelihood ratio (PLR), negative likelihood ratio (NLR), positive predictive value (PPV), and negative predictive value (NPV) of the scoring system in the training set were 87.8%, 92.6%, 11.8, 0.13, 89.3%, and 91.5%, respectively (**Table 4**). The accuracy of the prediction score for differentiating MPE from BPE in the internal and external validation sets is shown in **Table 4**, and the ROC curves are presented in **Figure 3A**. The AUCs of 0.955 (95% CI = 0.942–0.968) for the training set, 0.952 (95% CI = 0.932–0.973) for the internal validation set, and 0.973 (95% CI = 0.956–0.990) for the external validation set indicated that the scoring system showed good discriminative power in differentiating between MPE and BPE. Moreover, the calibration curve of the scoring system also depicted good agreement between the observed and predicted probabilities in all three datasets (**Figures 3B–D**).

**Table 3:** Diagnostic nomogram score calculation.

Parameters	Points
ESR (≤43)	3
Effusion CEA (>5)	10
Effusion CA19-9 (>9.2)	4
Effusion CYFRA21-1 (>59.6)	4
Effusion/Serum CEA (>1.14)	5
Effusion ADA (≤25)	5
Serum LDH/effusion ADA (>9.4)	5

ESR, erythrocyte sedimentation rate; CEA, serum carcinoembryonic antigen; CA19-9, carbohydrate antigen 19-9; CYFRA21-1, cytokeratin 19 fragment; LDH, lactate dehydrogenase.

**Table 4:** Accuracy of the prediction score of the nomogram for differentiating MPE from BPE.

Variables	MPE/BPE		
	Training set	Internal validation set	External validation set
AUC (95% CI)	0.955 (0.942–0.968)	0.952 (0.932–0.973)	0.973 (0.956–0.990)
Sensitivity (95% CI)	87.8% (83.9–90.9)	85.1% (77.6–90.4)	89.5% (80.6–84.8)
Specificity (95% CI)	92.6% (89.9–94.6)	91.9% (87.2–95.1)	92.4% (86.1–96.1)
PLR (95% CI)	11.8 (8.72–16.0)	10.6 (6.66–16.7)	11.7 (6.44–21.4)
NLR (95% CI)	0.13 (0.10–0.173)	0.16 (0.11–0.24)	0.11 (0.06–0.21)
PPV (95% CI)	89.3% (85.5–92.2)	87.0% (79.8–92.0)	88.5% (79.4–94.1)
NPV (95% CI)	91.5% (88.7–93.7)	90.7% (85.7–94.1)	93.1% (86.9–96.6)

MPE, malignant pleural effusion; BPE, benign pleural effusion; AUC, area under curve; CI, confidence interval; PLR, positive likelihood ratio; NLR, negative likelihood ratio; PPV, positive predictive value; NPV, negative predictive value.

### Diagnostic Performance of the Scoring System for Identifying Lung Cancer-Associated MPE and TPE

**Figure 4:**
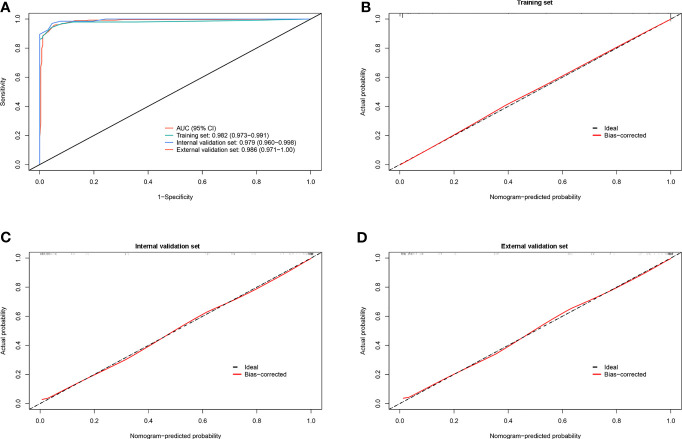
Discrimination and calibration of the scoring system for identifying lung cancer with pleural effusion (PE) from tuberculous pleurisy effusion (TPE). **(A)** Receiver operating characteristic (ROC) curves of the scoring system in the training, internal validation, and external validation sets. **(B–D)** Calibration plots of the scoring system in the training, internal validation, and external validation sets.

To further investigate the potential impact of the scoring system on the discriminative ability to separate lung cancer-associated MPE from TPE, we set the optimal cutoff as 15 points, which might accelerate clinical decision-making. The scoring system achieved good discriminatory efficacy in the combined training and validation sets and yielded AUCs of 0.982 (95% CI = 0.973–0.991), 0.979 (95% CI = 0.960–0.998), and 0.986 (95% CI = 0.971–1.0) (**Figure 4A**). The calibration curve of the scoring system did not show any deviation from the perfect fit in the training and validation sets (**Figures 4B–D**). The statistical data for sensitivity, specificity, PLR, NLR, PPV, and NPV are summarized in **Table 5**.

**Table 5:** Accuracy of the prediction score of the nomogram for differentiating lung cancer-associated MPE from TPE.

Variables	Lung cancer-associated MPE/TPE		
	Training set	Internal validation set	External validation set
AUC (95% CI)	0.982 (0.973–0.991)	0.979 (0.960–0.998)	0.986 (0.971–1.00)
Sensitivity (95% CI)	90.4% (86.1–93.4)	86% (77.3–91.9)	92.5% (82.7–97.2)
Specificity (95% CI)	96.6% (93.4–98.3)	99.0% (0.94–100)	95.5% (86.4–98.8)
PLR (95% CI)	26.6 (14.0–50.6)	82.6 (11.7–581.1)	20.4 (6.72–61.6)
NLR (95% CI)	0.10 (0.07–0.14)	0.14 (0.09–0.23)	0.08 (0.03–0.18)
PPV (95% CI)	96.6% (93.4–98.3)	98.9% (92.9–1)	95.4% (86.2–98.8)
NPV (95% CI)	90.5% (86.3–93.5)	87.2% (79.1–92.5)	92.6% (83–97.3)

MPE, malignant pleural effusion; TPE, tuberculous pleurisy effusion; AUC, area under curve; CI, confidence interval; PLR, positive likelihood ratio; NLR, negative likelihood ratio; PPV, positive predictive value; NPV, negative predictive value.

## Discussion

PE is a common clinical manifestation caused by more than 50 diseases. Tuberculosis, pneumonia, and malignancy are among the most common causes of PE (2, 3, 11). In recent years, patients with BPE, such as TPE and PPE, can be clinically cured due to advances in treatment regimens and early diagnosis (2, 22). Conversely, MPE usually indicates advanced malignant diseases related to heavy healthcare burden and a high mortality rate. Early diagnosis and selection of individualized therapy are critical to improving the outcomes of patients with MPE (23). The primary diagnostic methods of MPE are thoracentesis, cytology, and histology (12, 15). However, the invasive intervention, high cost, or the low accuracy associated with these conventional methods are inadequate for all patients with MPE. Thus, a more effective and less invasive diagnostic tool is an urgent requirement to improve the categorization and management of MPE.

A large number of studies have attempted to discriminate between MPE and BPE over the past decades. Daniel et al. (24) established a CELLSEARCH system based on circulating tumor cell detect technology to diagnose MPE. The system showed excellent performance with an AUC of 0.86 for all effusions. Yang et al. (25) developed a scoring system based on five PET-CT parameters in diagnosing MPE, which achieved a sensitivity of 83.3% and a specificity of 92.2% in the derivation set. Moreover, Nakamura et al. (26) applied next-generation sequencing (NGS) of single-cell sequencing technology to diagnose primary and metastatic tumors from PE. This method overcame the issue of low pleural volume, thereby expanding the possibility of personalized clinical treatment. Despite significant advances in understanding the etiology of PE, the above methods are not suitable and available in most primary facilities due to their high cost and requirement of expensive and sophisticated medical equipment. Thus, developing a method to accurately differentiate MPE from BPE at the earliest is highly desirable.

The utility of biomarkers in the diagnosis of MPE has been studied extensively (16, 18). Nonetheless, which biomarkers are the key to determining the diagnosis of MPE are yet controversial. The traditional biomarkers, including CEA, CA125, CA19-9, CA15-3, CYFRA 21-1, and NSE, could be valuable in MPE diagnosis, whereas their low sensitivity and/or specificity render limited merit for definitive diagnosis (27–29). Furthermore, some ratios such as those of effusion/serum CEA, serum LDH/effusion ADA, and effusion NC/LC might also be useful in predicting MPE, but the optimal cutoff values are not yet recommended due to the heterogeneity of the test methods (17, 21, 30). Nonetheless, these biomarkers are cost-effective, noninvasive, readily available, and may offer valuable information for diagnosis. Previous studies have attempted to use various parameters to predict MPE, but lacked validation due to limited sample sizes (28, 30, 31). In the present study, we initially integrated 38 variables, including not only primary clinical and laboratory variables but also calculated ratios. Among the currently available prediction approaches, a nomogram based on multiple markers has both high accuracy and good discrimination characteristics in the diagnostic performance and is convenient for clinical use (32). The proposed nomogram in our study incorporated the seven most significant variables (ESR, effusion CEA, effusion CA19-9, effusion CYFRA21-1, CEA ratio, effusion ADA, and CR) and provided favorable calibration and discrimination in both derivation and validation sets.

Some studies have applied an artificial intelligence framework for the diagnosis of MPE and BPE; such models require specific software that would promote their wide application (33, 34). Herein, we modified the nomogram into a scoring system for clinical application. Patients with a score of more than 15 are more likely to be diagnosed as MPE. This simple and feasible scoring system with high reliability discriminated MPE from BPE in the training, internal validation, and external validation sets. The accuracy of the scoring system was also estimated by the sensitivity, specificity, PLR, NLR, PPV, and NPV in the training and validation sets. The encouraging results supported that the scoring system is a quantitative and valuable tool in discriminating MPE from BPE.

Lung cancer-associated MPE and TPE are the most common exudative effusions in China (30, 35, 36). Since these diseases are predominantly lymphocytic and have similar clinical manifestations, the clinical differentiation between the two effusions is challenging. In the present study, we applied the scoring system to diagnose between the two diseases and achieved good discriminatory efficacy in the combined training and validation sets. The high sensitivity and specificity also indicated that this scoring system is useful in distinguishing lung cancer-associated MPE from TPE. The scoring system comprehensively integrated clinical and laboratory indicators, which might be better than any single parameter alone. A previous study reported a scoring system based on a nomogram that incorporated six factors (fever, ESR, ADA, effusion CEA, serum CEA, and CEA ratio) and obtained a favorable diagnostic performance in distinguishing lung cancer-associated MPE from TPE (19). Also, other tumor markers have been reported to be useful in lung cancer (such as CYFRA 21-1, NSE, or CR), which were not evaluated in this study. In addition, novel biomarkers, such as serum interleukin-27 (IL-27), gas chromatography–mass spectrometry (GC-MS) metabolomics, and serum reactive oxygen species modulator 1 (ROMO1), have been proposed as potential indexes for diagnosing lung cancer-associated MPE, but only a few were routinely detected in clinical practice (18, 37–39).

To the best of our knowledge, the main strengths of the present investigation are the large sample size and involvement of the major laboratory indexes. The established scoring system based on seven easily accessible and inexpensive clinical parameters might be suitable for routine clinical practice in most hospitals. However, the current study has several limitations. Firstly, we could not eliminate the inherent bias due to its retrospective nature. Secondly, external validation in a large cohort and multiple institutions is needed to confirm the performance of the scoring system. Finally, the presented scoring system did not include radiological and epigenetic indicators, which might be valuable to improving the diagnostic accuracy of MPE. Thus, additional studies are warranted to address these issues.

## Conclusion

The current study developed and validated a scoring system based on seven easily accessible parameters (ESR, effusion CEA, effusion CA19-9, effusion CYFRA21-1, CEA ratio, effusion ADA, and CR). This scoring system showed good calibration and diagnostic performance in distinguishing MPE from BPE. The scoring system also achieved good discriminatory efficacy in discriminating between lung cancer-associated MPE and TPE.

## Data Availability Statement

The raw data supporting the conclusions of this article will be made available by the authors, without undue reservation.

## Ethics Statement

The studies involving human participants were reviewed and approved by the Institutional Ethics Committee of Ningbo First Hospital and the Institutional Ethics Committee of The Affiliated People Hospital of Ningbo University. The Ethics Committee waived the requirement of written informed consent for participation.

## Author Contributions

AW and YL were responsible for the study concept and design and critical revision of the manuscript for important intellectual content. ZL, WP, JY, and SW contributed to data collection, analysis, and validation. SY and YM helped with data interpretation and drafted the manuscript. All authors contributed to the article and approved the submitted version.

## Funding

This work was supported by the Open Project of Key Laboratory of Zhejiang Province (grant no. AMSMAKF2102).

## Conflict of Interest

The authors declare that the research was conducted in the absence of any commercial or financial relationships that could be construed as a potential conflict of interest.

## Publisher’s Note

All claims expressed in this article are solely those of the authors and do not necessarily represent those of their affiliated organizations, or those of the publisher, the editors and the reviewers. Any product that may be evaluated in this article, or claim that may be made by its manufacturer, is not guaranteed or endorsed by the publisher.

## Supplementary Material

Supplementary Figure 1 Diagnostic ability and calibration of the nomogram for identifying MPE from BPE. The ROC curves of the nomogram in the training, internal validation and external validation sets, respectively **(A)**. Calibration curves of the nomogram in the training, internal validation and external validation sets, respectively **(B–D)**.
