# Supplementary figures and images for: Development and Validation of a Scoring System for Early Diagnosis of Malignant Pleural Effusion Based on a Nomogram
Source: Front Oncol. 2021 Dec 7;11:775079. doi: 10.3389/fonc.2021.775079 (PMC8688822; doi:10.3389/fonc.2021.775079)

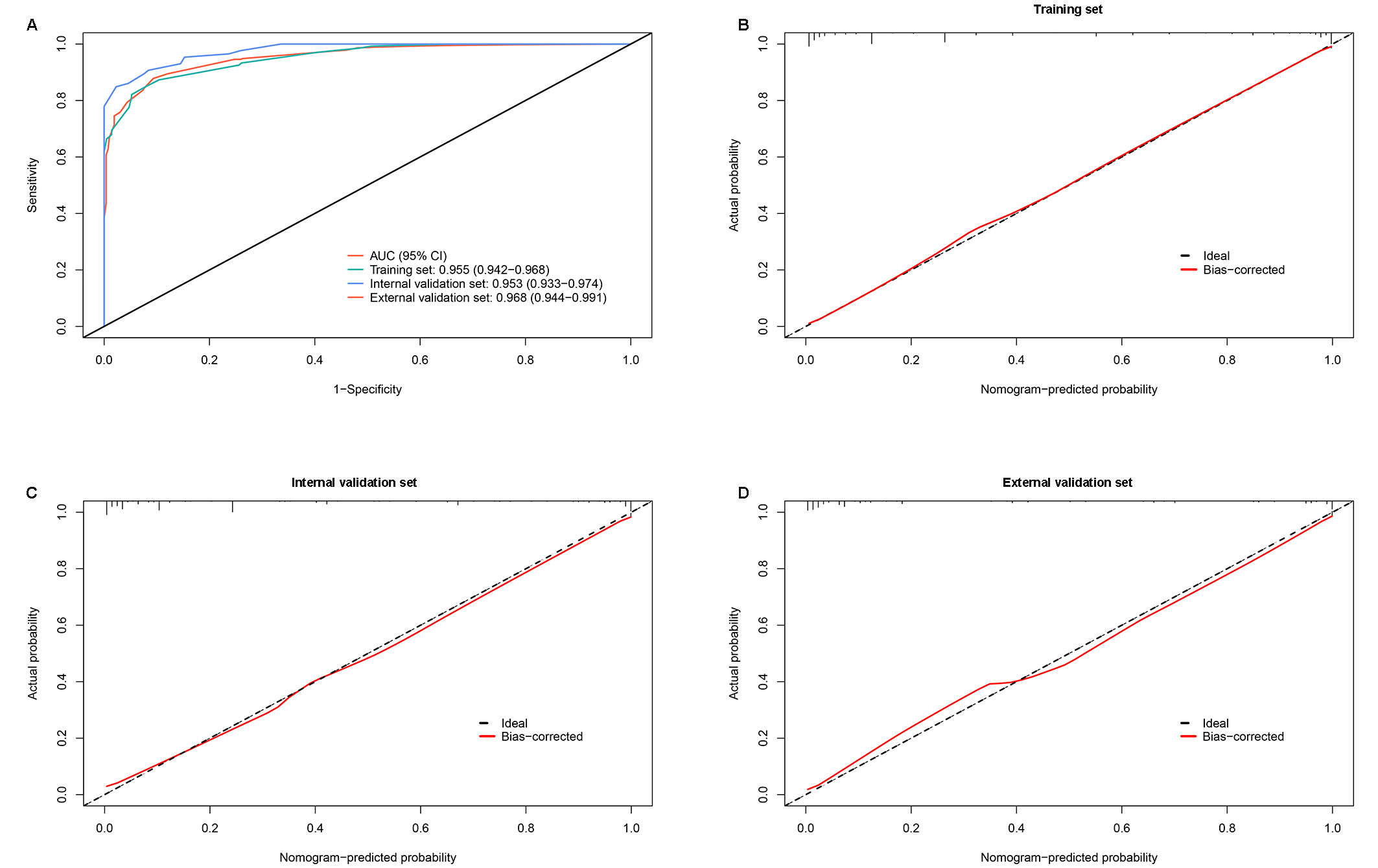

Supplement: Supplementary Figure 1 — Diagnostic ability and calibration of the nomogram for identifying MPE from BPE. The ROC curves of the nomogram in the training, internal validation and external validation sets, respectively (A). Calibration curves of the nomogram in the training, internal validation and external validation sets, respectively (B–D). [file Image_1.tif]
