# Supplementary material for: Development and Validation of a Scoring System for Early Diagnosis of Malignant Pleural Effusion Based on a Nomogram
Source: Front Oncol. 2021 Dec 7;11:775079. doi: 10.3389/fonc.2021.775079 (PMC8688822; doi:10.3389/fonc.2021.775079)
Supplement: Supplementary file 2 [file Table_1.docx]

**Supplementary Table 1.** Clinical characteristics of the patients with PE in the training cohort

| Parameters | BPE (n=526) | MPE (n=368) | *p* value |
| --- | --- | --- | --- |
| Age(years) | 56.17 ± 20.43 | 67.16 ± 12.71 | < 0.0001 |
| Gender |  |  |  |
| Male | 357 | 216 | 0.003 |
| Female | 169 | 152 |  |
| Smoking history |  |  |  |
| Yes | 184 | 152 | 0.062 |
| No | 342 | 216 |  |
| Serum WBC (10^9^/L) | 7.46±3.20 | 7.91±3.33 | 0.049 |
| Serum NC (10^9^/L) | 5.50±3.56 | 5.77±3.05 | 0.223 |
| Serum LC (10^9^/L) | 1.17±0.55 | 1.34±1.22 | 0.004 |
| CRP | 57.02 ± 59.40 | 26.66 ± 38.58 | < 0.0001 |
| Serum ADA | 11.53±7.64 | 12.53±7.26 | 0.048 |
| Serum LDH | 209.18±97.19 | 236.71±218.72 | 0.011 |
| ESR | 47.24±26.59 | 35.66±26.24 | < 0.0001 |
| Serum CEA | 1.86±1.27 | 73.21±197.63 | < 0.0001 |
| Serum CA125 | 139.29±147.29 | 255.85±604.73 | < 0.0001 |
| Serum CA19-9 | 10.46±16.43 | 108.93±352.12 | < 0.0001 |
| Serum CYFRA21-1 | 2.48±4.46 | 9.35±16.11 | < 0.0001 |
| Serum NSE | 13.09±8.43 | 20.81±32.59 | < 0.0001 |
| Effusion WBC | 4.44±20.93 | 3.04±17.35 | 0.292 |
| Effusion N% | 24.05±26.96 | 23.61±22.58 | 0.797 |
| Effusion L% | 63.95±30.21 | 58.84±26.52 | 0.01 |
| Effusion TP | 44.4±13.88 | 45.21±10.21 | 0.339 |
| Effusion Glu | 6.08±3.08 | 6.61±3.10 | 0.012 |
| Effusion ADA | 32.27±28.26 | 14.21±25.41 | < 0.0001 |
| Effusion LDH | 497.76±534.39 | 1190.4±10952 | 0.148 |
| Effusion CEA | 1.56±3.73 | 282.26±401.27 | < 0.0001 |
| Effusion CA125 | 1316.64±1045.11 | 2086.53±1532.02 | < 0.0001 |
| Effusion CA19-9 | 9.19±95.24 | 292.71±606.23 | < 0.0001 |
| Effusion CYFRA21-1 | 37.13±52.98 | 151.96±178.63 | < 0.0001 |
| Effusion NSE | 14.73±41.62 | 32.69±62.35 | < 0.0001 |

PE, pleural effusion; BPE, benign pleural effusion; MPE, malignant pleural effusion; WBC, white blood cells; NC, neutrophil cells; LC, lymphocytes; CRP, C-reactive protein; TP, total protein; Glu, glucose; ADA, adenosine deaminase; LDH, lactate dehydrogenase; ESR, erythrocyte sedimentation rate; CEA, carcinoembryonic antigen; CA125, carbohydrate antigen 125; CA19-9, carbohydrate antigen 19-9; CYFRA21-1, cytokeratin 19 fragment; NSE, neuron-specific enolase.

**Supplementary Table 2.** Univariate logistic regression analysis of clinical parameters in the training cohort.

| Variable | OR (95% CI) | Wald | *p* value | AUC |
| --- | --- | --- | --- | --- |
| Age, years | 1.036 (1.028-1.045) | 70.51 | < 0.0001 | 0.641 |
| Gender, female vs male | 0.657 (0.498-0.866) | 8.869 | 0.003 | 0.549 |
| Smoking history, yes vs no | 1.298 (0.987-1.707) | 3.482 | 0.062 |  |
| Serum WBC, > 6.64 vs ≤ 6.64 (109/L) | 1.655 (1.257-2.179) | 12.89 | < 0.0001 | 0.562 |
| Serum NC, > 4.85 vs ≤ 4.85 (109/L) | 1.024 (0.984-1.065) | 1.35 | 0.245 |  |
| Serum LC, > 1.2 vs ≤ 1.2 (109/L) | 1.595 (1.213-2.096) | 11.21 | 0.001 | 0.558 |
| CRP, > 20 vs ≤ 20 mg/L | 0.261 (0.197-0.346) | 86.96 | < 0.0001 | **0.662** |
| Serum ADA, > 22 vs ≤ 22 U/L | 1.441 (0.829-2.506) | 1.678 | 0.195 |  |
| Serum LDH, > 250 vs ≤ 250 U/L | 1.084 (0.768-1.532) | 0.211 | 0.646 |  |
| ESR, > 43 vs ≤ 43 mm/h | 0.229 (0.172-0.306) | 99.98 | < 0.0001 | **0.674** |
| Serum CEA, > 5 vs ≤ 5 ng/mL | 41.81 (23.24-75.20) | 155.31 | < 0.0001 | **0.745** |
| Serum CA125, > 110 vs ≤ 110 U/mL | 1.199 (0.913-1.576) | 1.703 | 0.192 |  |
| Serum CA199, > 25 vs ≤ 25 ng/mL | 8.505 (5.414-13.36) | 86.31 | < 0.0001 | 0.627 |
| Serum SYFRA21-1, > 3.3 vs ≤ 3.3 ng/mL | 9.40 (6.882-12.83) | 198.76 | < 0.0001 | **0.747** |
| Serum NSE, > 16.3 vs ≤ 16.3 ng/mL | 2.87 (2.097-3.928) | 43.36 | < 0.0001 | 0.60 |
| Effusion WBC, > 2.25 vs ≤ 2.25 (10^9^/L) | 0.352 (0.258-0.482) | 42.52 | < 0.0001 | 0.609 |
| Effusion N%, > 7 vs ≤ 7 | 1.279 (0.955-1.713) | 2.73 | 0.098 |  |
| Effusion L%, > 85.5 vs ≤ 85.5 | 0.516 (0.376-0.707) | 16.86 | < 0.0001 | 0.565 |
| Effusion TP, > 50 vs ≤ 50 g/L | 0.609 (0.459-0.807) | 11.87 | 0.001 | 0.559 |
| Effusion Glu, > 5.8 vs ≤ 5.8 mmol/L | 1.988 (1.502-2.631) | 23.08 | < 0.0001 | 0.583 |
| Effusion ADA, > 25 vs ≤ 25 U/L | 0.089 (0.061-0.128) | 166.23 | < 0.0001 | **0.737** |
| Effusion LDH, > 300 vs ≤ 300 U/L | 0.848 (0.644-1.116) | 1.38 | 0.24 |  |
| Effusion CEA, > 5 vs ≤ 5 ng/mL | 83.75(47.11-148.9) | 227.44 | < 0.0001 | **0.835** |
| Effusion CA125, > 2050 vs ≤ 2050 U/mL | 3.38 (2.483-4.594) | 60.11 | < 0.0001 | 0.621 |
| Effusion CA19-9, > 9.2 vs ≤ 9.2 ng/mL | 10.43 (7.278-14.96) | 162.82 | < 0.0001 | **0.710** |
| Effusion CYFRA21-1, > 59.6 vs ≤ 59.6 ng/mL | 6.56 (4.787-9.0) | 136.44 | < 0.0001 | **0.693** |
| Effusion NSE, > 15.8 vs ≤ 15.8 ng/mL | 2.964 (2.192-4.007) | 49.88 | < 0.0001 | 0.611 |
| Effusion/Serum CEA, > 1.14 vs ≤ 1.14 | 6.98 (5.093-9.566) | 145.98 | < 0.0001 | **0.701** |
| Effusion/Serum CA125, > 11.2 vs ≤ 11.2 | 1.743 (1.323-2.292) | 15.80 | < 0.0001 | 0.569 |
| Effusion/Serum CA199, > 0.77 vs ≤ 0.77 | 6.79 (4.927-9.357) | 137.03 | < 0.0001 | **0.692** |
| Effusion/Serum CYFRA21-1, > 53 vs ≤ 53 | 2.042 (1.366-3.051) | 12.112 | 0.001 | 0.54 |
| Effusion/Serum NSE, > 1.0 vs ≤ 1.0 | 1.79 (1.347-2.379) | 16.13 | < 0.0001 | 0.566 |
| Effusion/Serum LDH, > 2.8 vs ≤ 2.8 | 0.78 (0.583-1.037) | 2.93 | 0.087 |  |
| Effusion/Serum ADA, > 1.67 vs ≤ 1.67 | 0.089 (0.062-0.127) | 175.72 | < 0.0001 | **0.744** |
| Effusion/Serum WBC, > 0.4 vs ≤ 0.4 | 0.301 (0.214-0.422) | 48.17 | < 0.0001 | 0.613 |
| Serum NC/LC, > 5.9 vs ≤ 5.9 | 0.92 (0.685-1.236) | 0.303 | 0.582 |  |
| Effusion NC/LC, > 0.15 vs ≤ 0.15 | 1.73 (1.321-2.288) | 15.55 | < 0.0001 | 0.566 |
| Serum LDH/Effusion ADA, > 9.4 vs ≤ 9.4 | 11.00 (7.634-15.84) | 165.66 | < 0.0001 | **0.736** |

WBC, white blood cells; NC, neutrophil cells; LC, lymphocytes; CRP, C-reactive protein; TP, total protein; Glu, glucose; ADA, adenosine deaminase; LDH, lactate dehydrogenase; ESR, erythrocyte sedimentation rate; CEA, carcinoembryonic antigen; CA125, carbohydrate antigen 125; CA19-9, carbohydrate antigen 19-9; CYFRA21-1, cytokeratin 19 fragment; NSE, neuron-specific enolase; OR, odds ratio; AUC, area under curve; CI, confidence intervals.
